# Detection of circulating hepatitis B virus immune escape and polymerase mutants among HBV-positive patients attending Institut Pasteur de Bangui, Central African Republic

**DOI:** 10.1016/j.ijid.2019.10.039

**Published:** 2019-11-01

**Authors:** Giscard Wilfried Koyaweda, Juliette Rose Ongus, Eunice Machuka, John Juma, Rosaline Macharia, Narcisse Patrice Komas, Roger Pelle

**Affiliations:** a Pan African University, Institute for Basic Sciences Technology and Innovation, Nairobi, Kenya; b Jomo Kenyatta University of Agriculture and Technology, Medical Laboratory Sciences Department, Nairobi, Kenya; c Biosciences eastern and central Africa International Livestock Research Institute (BecA-ILRI) Hub, Nairobi, Kenya; d Center for Biotechnology and Bioinformatics, University of Nairobi, Nairobi, Kenya; e Institut Pasteur de Bangui, Viral Hepatitis Laboratory, Bangui, Central African Republic

**Keywords:** Hepatitis B virus, Genotype/subgenotype, Serotype, Immune escape mutants (IEMs), Polymerase mutants, Central African Republic

## Abstract

**Background:**

Previous studies in the Central African Republic (CAR) have reported the presence of hepatitis B virus (HBV) recombinant genotype E/D and a suspicion of immune escape mutants (IEMs), without further investigation into their impact on prevention and diagnosis. Consequently, this study investigated HBV mutations among hepatitis B surface antigen (HBsAg)-positive patients attending Institut Pasteur de Bangui in the CAR.

**Methods:**

Sera from a total of 118 HBsAg-positive patients with no previous history of HBV treatment or vaccination at the Institut Pasteur de Bangui, were sampled between 2017 and 2019. Subsequently, the region spanning the surface and polymerase genes of HBV was amplified by PCR and sequenced. HBV sequences were genotyped/subgenotyped by phylogenetic analysis and serotyped based on predicted amino acid residues at positions s122, s127, s140, s159, and s160. They were then analyzed for HBV IEMs and polymerase mutations.

**Results:**

The region spanning the surface and polymerase genes was successfully amplified and sequenced for 51 samples. Of the HBV sequences, 49 were genotype E and two were genotype A subgenotype A1; these were serotyped as ayw4 and ayw1, respectively. Potential IEMs sY100C, sA128V, and sM133T, and several polymerase mutants were identified.

**Conclusions:**

This study raises awareness of the need for further studies to be conducted on a large scale to better understand HBV mutations for improved disease control and prevention strategies in the country.

## Introduction

Hepatitis B virus (HBV) infection remains a serious health problem worldwide. Globally, HBV infection is one of the most dangerous infectious diseases, causing significant mortality (Mokaya et al., 2018). Infection by HBV results in inflammation of the liver. The infection can progress from acute hepatitis to chronic infection. The World Health Organization (WHO) estimated that in the year 2015, 257 million people worldwide were living with chronic HBV (CHB) infection, and 68% of those infected were living in the African and Western Pacific regions (WHO, 2017). This estimation showed that the second largest number of chronically infected HBV patients are living in the African continent, with 6.1% of the adult population infected (WHO, 2017).

In the Central African Republic (CAR), a previous study reported a high prevalence of HBV in rural areas, with overall hepatitis B core antibody (HBc antibody) prevalence of 27.1% and hepatitis B surface antigen (HBsAg) prevalence of 10.6% (Komas et al., 2013). This finding highlights the possible impact of HBV on the economy of CAR, because these regions are the key farming regions.

The United Nations Sustainable Development Goals set out the challenge of the elimination of viral hepatitis as a public health threat by the year 2030 (WHO, 2016). The most effective measure to reduce the global incidence of hepatitis B remains vaccination. A mathematical model for the estimation of the global incidence of HBV showed that without vaccination, 64.8 million of the surviving birth cohort for the year 2000 would be infected by HBV and 1.4 million would die because of HBV complications (Goldstein et al., 2005).

HBV is categorized into eight genotypes, A-H, based on divergence of more than 8% in the entire nucleotide sequence of the viral DNA; two additional genotypes I and J have also been proposed (Lin and Kao, 2011; Kramvis, 2014). There are also subgenotypes with 4-8% genomic variability. The HBV genotypes show a distinct geographical distribution worldwide, and genotypes A, D, and E are the most frequently found in Africa. In Western and Central Africa, genotype E is the dominant strain (Kramvis, 2014). A previous study in the CAR reported a prevalence of 94% genotype E, 4.5% genotype D, and 1.5% subgenotype A1 (Bekondi et al., 2007). In 2013, 100% of 19 isolated HBV were identified as genotype E (Komas et al., 2013).

Although the genome of HBV is made up of DNA, the virus transitions to an unstable RNA state during replication, where its proof-reading deficient reverse transcriptase does not correct errors. This results in the accumulation of mutations. Mutations in the HBV genome of each genotype may affect prevention strategies, the diagnosis techniques, the response to treatment, and the course of the disease (Mokaya et al., 2018). Mutations in HBV that are associated with vaccine escape and detection escape have been documented in many countries worldwide; however, these remain poorly documented in some African countries (Mokaya et al., 2018). In the CAR, there is a lack of robust information in regards to HBV vaccine escape mutations and the accumulation of polymerase mutations. Previous studies in the country have reported the presence of recombinant genotype E/D (Bekondi et al., 2007) and a suspicion of vaccine escape mutants (Komas et al., 2013), without further investigation into their impact on the prevention and diagnosis measures in place.

## Materials and methods

### Study site and sample collection

This study was conducted at Institut Pasteur de Bangui in the CAR, a landlocked country in Central Africa. Serum samples were collected both retrospectively and prospectively between the years 2017 and 2019 in the Viral Hepatitis Laboratory, Institut Pasteur de Bangui. All patients included in this study had no previous history of any vaccination or treatment for HBV, as seen in their records at the institute. For the archived samples, the medical information of the patients was used to collect only confirmed HBsAg-positive samples. Fifty-three samples were collected from the archive (years 2017-2018). Older samples had been discarded from the institute to allow a new archiving process. The remaining 75 samples were collected prospectively from January to February 2019. All of the patients who were positive for HBsAg on screening and who were also confirmed to be HBsAg-positive had samples collected immediately and stored at –20°C. The total number of samples collected was 118. All of the patients included in this study came to the institute for HBV screening and were enrolled after being consented.

### HBV serological assays

An ELISA (Abbott-Murex kit, United Kingdom) for the detection of HBsAg marker was performed for all samples, in accordance with the manufacturer’s instructions. HBsAg-positive samples were confirmed by Murex HBsAg confirmatory test version 3 and tested for hepatitis B e antigen (HBeAg) using the Creative Diagnostic Human HBeAg ELISA Kit (catalog number DEIA003). Confirmed HBsAg-positive samples were screened for hepatitis delta virus (HDV) using a DiaSorin kit (DiaSorin Italy). All results were interpreted in accordance with the manufacturers’ instructions.

### Viral DNA extraction

Viral hepatitis DNA was extracted from 200 ml of serum using the QIAamp DNA Mini Kit (catalog number 51306; Qiagen, Germany) according to the manufacturer’s instructions.

### Amplification of the surface and polymerase genes

The primer pair Pol forward and reverse was used, as described previously (Sayan et al., 2010), to amplify a 742-bp DNA fragment spanning the surface and polymerase gene regions (Table 1). In brief, the PCR mix was carried in a total volume of 50 µl with a final concentration of 0.1 pmol of each primer, 2.5 U of *Taq* DNA polymerase, 250 µM of each dNTP, 10 mM of Tris-HCl (pH 9.0), 30 mM of KCl, 1.5 mM of MgCl_2_, stabilizer and tracking dye, and 0.8-2 ng/µl of DNA template. A ProFlex PCR Thermal Cycler (Applied Biosystems) was used for thermal cycling, as follows: 95 °C for 5 min, and then 45 cycles consisting of 95 °C for 45 s, 56 °C for 45 s, and 72 °C for 45 s. A final elongation was set at 72 °C for 10 min.

**Table 1:** List of primers used in the study for PCR amplification and sequencing.

Primer	Sequence	Used	Region	Product size (bp)	Reference
Pol F	5′ TCGTGGTGGACTTCTCTCAATT 3′	PCR and sequencing	Polymerase	740	Sayan et al. (2010)
Pol R	5′ CGTTGACAGACTTTCCAATCAAT 3′	PCR and sequencing	Polymerase	740	Sayan et al. (2010)
WA-L	*5′* ACTGTTCAAGCCTCCAAGCTGTGC 3′	PCR	Whole genome	3200	Zhang et al. (2007)
WA-R	5^′^ AGCAAAAAGTTGCATGGTGCTGGT 3^′^	PCR	Whole genome	3200	Zhang et al. (2007)
A3-L	5^′^ CTGCTGGTGGCTCCAGTT 3^′^	Sequencing	Polymerase	1059	Zhang et al. (2007)
A3-R	5^′^ GCCTTGTAAGTTGGCGAGAA 3^′^	Sequencing	Polymerase	1059	Zhang et al. (2007)
A4-L	5^′^ GTATTGGGGGCCAAGTCTGT 3^′^	Sequencing	Polymerase	1072	Zhang et al. (2007)
A4-R	5^′^ AAAAAGTTGCATGGTGCTG 3^′^	Sequencing	Polymerase	1072	Zhang et al. (2007)

### Amplification of the HBV whole genome

In an additional investigation, the complete genome (3.2 kb) of one sample, for which the region spanning the surface and polymerase gene was successfully sequenced in the present study, was amplified with the primers WA-L and WA-R, as described previously (Zhang et al., 2007). In brief, the PCR mix was prepared as described for the surface and polymerase gene PCR, using the primer pair WA-L and WA-R (Table 1). The ProFlex PCR Thermal Cycler (Applied Biosystems) was used for thermal cycling, as follows: 95 °C for 5 min, and then 30 cycles consisting of 95 °C for 30 s, 58 °C for 1 min, and 72 °C for 3 min 30 s. A final elongation was set at 72 °C for 10 min.

### Purification and sequencing of PCR products

All PCR products were resolved on 1% agarose gels stained with GelRed and viewed using a UV transilluminator. Sanger sequencing was performed by Macrogen (Netherlands). The fragment spanning the surface and polymerase gene regions was sequenced using the same PCR primer pair, and the PCR product of the whole genome was sequenced with a couple of overlapping primers A3-L/ A3-R and A4-L/A4-R, as described previously (Zhang et al., 2007), to generate the sequence of the entire reverse transcriptase of HBV (Table 1).

### Sequence cleaning up and assembly

All of the sequences were analyzed and assembled using CLC Genomic Workbench 8.0.3 (https://www.qiagenbioinformatics.com/blog/discovery/publications-citing-clc-genomics-work-bench/) and then subjected to NCBI nucleotide BLAST for quality check.

### Sequence genotyping/subgenotyping and serotyping

A phylogenetic analysis was done using MEGA version 10.0.5. (Kumar et al., 2018). The analysis was done using the neighbor-joining statistical method, the Kimura-2 parameter model, and the bootstrap method of 1000 replicates. Genotypes and subgenotypes were confirmed with geno2pheno HBV (https://hbv.geno2pheno.org/index.php) and the genotyping tool of NCBI (https://www.ncbi.nlm.nih.gov/projects/genotyping/formpage.cgi).

Serotyping was done based on amino acids at positions s122, s127, s140, s159, and s160 (Swenson et al., 1991; Bell and Kramvis, 2015) by aligning the surface antigen amino acid sequence of 51 isolates against the reference sequences (gnl|hbvcds|AB014370 genotype A and gnl|hbvcds|AB091255 genotype E) in BioEdit.

### Analysis of mutations in HBsAg and polymerase

The overlapping surface (S) and polymerase gene sequences obtained were translated to the protein sequences and aligned with the reference sequence **AB014370** for genotype A and **AB091255** for genotype E in BioEdit, for the analysis of mutations. Subsequently, sequences were submitted to geno2pheno HBV for mutations analysis confirmation. Amino acid exchanges in each sequence were recorded and searched in the scientific literature.

### Nucleotide sequence accession numbers

The sequences of the regions spanning the surface and polymerase gene reported in this study have been submitted to GenBank and assigned the accession numbers **MN047437** and **MN420406** to **MN420455**.

Accession numbers of HBV sequences retrieved from the HBV database are mentioned in each analysis and those used for phylogenetic analysis are shown in the phylograms.

### Ethical approval

The Scientific Committee of the Faculty of Medicine, University of Bangui, CAR, approved the study protocol (Ref. No. 21/UB/FACSS/ CSCVPER/19).

## Results

### General patient data

All 118 samples included in the study were confirmed to be HBsAg-positive by ELISA. Patients were between 14 and 64 years old, with approximately 68 of the 118 patients aged between 25 and 60 years (Table 2). By year, 64% of samples were collected in the year 2019, 28% in 2018, and 8% in 2017. There were more male patients (*n* = 84, 71%) than female patients (*n* = 34, 29%); the male to female sex ratio was 2.47. The serological tests showed that 23/118 (19%) patients were co-infected with HDV and 16/118 (14%) patients were HBeAg-positive.

**Table 2:** Age and sex distribution of the HBsAg-positive study population and distribution of patients positive for HBeAg and HDV; Institut Pasteur de Bangui 2017-2019.

Ages groups (years)	Male			Female			Total
	HBsAg frequency (%)	HBeAg-positive	HDV-positive	HBsAg frequency (%)	HBeAg-positive	HDV-positive	HBsAg frequency
>18	7 (5.93)	2	1	0 (0.0)	0	0	7
18-24	4 (3.39)	1	1	4 (3.39)	1	1	8
25-40	49 (41.52)	7	8	19 (16.1)	2	3	68
41-60	22 (18.64)	1	4	11 (9.32)	1	5	33
<60	2 (1.69)	0	0	0 (0.0)	0	0	2
Total	84	10	14	34	4	9	118

HBsAg, hepatitis B surface antigen; HBeAg, hepatitis B e antigen; HDV, hepatitis delta virus.

### PCR and sequencing

All 118 HBsAg-positive samples were subjected to viral DNA extraction. Subsequently, surface and polymerase genes were amplified by PCR and sequenced. Only 57/118 (48.30%) samples were PCR-positive and 51/57 PCR-positive samples were successfully sequenced; six of the PCR-positive samples gave sequences that were not exploitable.

The 51 patients whose samples were successfully sequenced were aged between 17 and 64 years; 69.05% were male and 30.95% were female, giving a male to female sex ratio of 2.4. Of these samples, 27.45% were positive for HBeAg and 13.72% were positive for co-infection with HDV. None of these patients had a history of treatment for HBV or history of HBV vaccination. Basic data of these 51 patients are given in Table 3.

**Table 3:** Basic data of the HBV patients whose samples were successfully sequenced (*n* = 51). Characteristics

Characteristics	Values
Sex (male:female; sex ratio)	29:13; 2.4
Age (years), mean (range)	37 (17-64)
HBeAg-positive, *n (%)*	14 (27.45)
HDV, n (%)	7 (13.72)
Vaccination	No record
Treatment	No record

HBV, hepatitis B virus; HBeAg, hepatitis B e antigen; HDV, hepatitis delta virus.

### HBV genotypes/subgenotypes and serotypes

**Figure 1:**
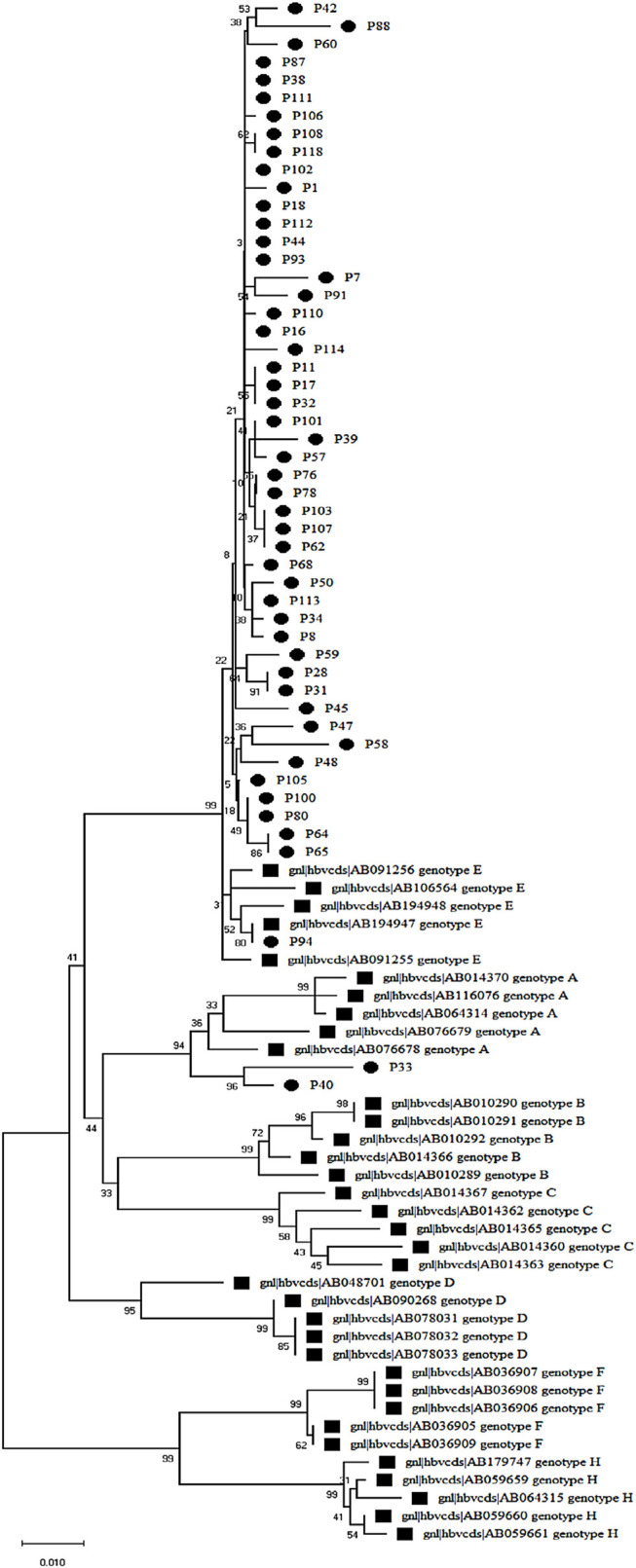
Phylogenetic tree of the partial polymerase (742 bp) of the HBV isolates. The tree was constructed in MEGA using the neighbor-joining statistical method, the Kimura-2 parameter model, and the bootstrap method of 1000 replicates. The black squares represent the reference sequences and the black circles represent the isolated HBV sequences discussed in the present study.

All 51 contig sequences of the isolated HBV showed significant similarity to the HBV sequences after being subjected to Nucleotide BLAST search on the NCBI BLAST webpage. The phylogram (Figure 1) of the 51 HBV isolates as well as sequences of the eight HBV genotypes A-H retrieved from the HBV database (http://hbvdb.ibcp.fr/HBVdb/) revealed that 49/51 (96.08%) belonged to genotype E and 2/51 (3.92%) belonged to genotype A, more specifically subgenotype A1, after being subjected to geno2pheno HBV and the genotyping tool of NCBI.

Using amino acid residues at positions s122, s127, s140, s159, and s160, the protein sequences of the 51 isolated HBV alongside the reference sequences of the HBV surface gene (gnl|hbvcds| AB014370 genotype A and gnl|hbvcds|AB091255 genotype E) showed that all 49 isolates previously genotyped as E were serotyped ayw4 and the two genotype A subgenotype A1 isolates were serotyped ayw1.

### Mutations identified in surface antigen

**Figure 2:**
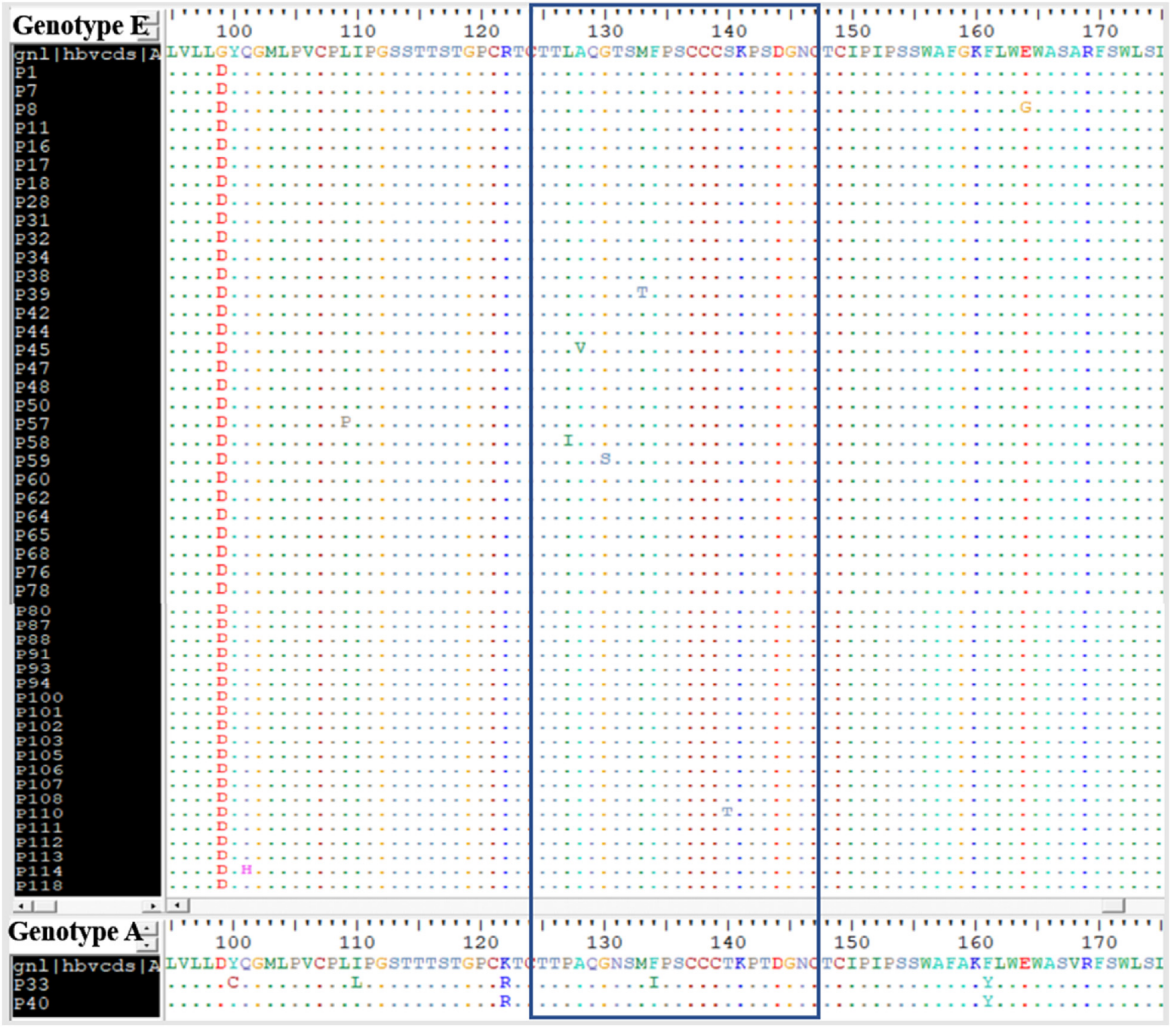
Mutation detected in the ‘a’ determinant. Genotype A isolates alongside a reference genotype A (GenBank reference **AB014370**) and genotype E isolates alongside a reference genotype E (GenBank reference **AB091255**). The box highlights the ‘a′ determinant region.

Alignment of the surface protein of the 51 isolated HBV alongside reference genotype A and E (Figure 2) and subsequent sequence submission to geno2pheno HBV on the web for analysis confirmation, led to the detection of 43 amino acid exchanges (Supplementary material). Six of these (sL127I, sA128V, sG130S, sM133T, sF134I, and S140T) were located in the ‘a’ determinant region, among which three immune escape mutants (IEMs) (sY100C, sA128V, and sM133T) were identified.

### Mutations identified in the polymerase gene

Alignment of the protein sequences of the partial polymerase gene of the 51 isolated HBV alongside reference genotype A and E, followed by sequence submission to geno2pheno HBV on the web for analysis confirmation, led to the detection of 43 amino acid exchanges (Supplementary material). These included the previously reported mutations rtL91I, rtM129L, rtW153R, rtS213T, rtV214A, rtN238T, rtN248H, and rtI269L (Yamani et al., 2017; Choi et al., 2018; Shaha et al., 2018; Ababneh et al., 2019). Of the 49 genotype E identified, 29 had amino acid exchanges in the polymerase gene. The two genotype A studied harbored 13 and 14 amino acid exchanges, respectively.

## Discussion

### HBV IEMs

This study is novel in investigating HBV escape mutations in the CAR. The study did not cover the entire CAR or Bangui populations, but was focused on individuals who were diagnosed at Institut Pasteur de Bangui from 2017 to 2019. This study detected three IEMs, sY100C, sA128V, and sM133T, similar to previous studies (Mello et al., 2011; Kwei et al., 2013; Cremer et al., 2018). Although data on IEMs in CAR are limited, the hypothesis of escape mutation among circulating HBV strains in the CAR was referred to previously in a study performed in 2013 (Komas et al., 2013). In that previous study, some individuals screened negative for HBsAg but positive for antibodies against HBc, with viral DNA detected in five of these individuals. The present study confirms this hypothesis. Therefore, there is the need for further investigations on HBV IEMs in the CAR.

sG145R is the most identified of the HBV IEMs and has been well-characterized, but was not identified in this study. The three IEMs identified in the present study (sY100C, sA128V, and sM133T) were confirmed by geno2pheno HBV. Mutation sY100C was previously assessed by Mello et al. (Mello et al., 2011), who showed a reduction in HBsAg detection level by commercial ELISA kit, but without statistical significance, leading the authors to conclude that mutation sY100C alone did not play a role in reducing the HBsAg affinity of this commercial ELISA assay. As suggested by Mello et al. (Mello et al., 2011), other mutations like K122R and F134I detected in the ‘a’ determinant support the idea of IEMs associated with strains harboring the sY100C as discussed in the present study. The mutation sA128V was confirmed by geno2pheno HBV, and this IEM was associated with vaccine escape in a recent study conducted by Cremer et al. (Cremer et al., 2018), who linked it with HBsAg test failure. Many studies (Protzer-Knolle et al., 1998; Beckebaum et al., 2003; Cheung et al., 2010) have demonstrated that M133T itself is frequently associated with occult HBV infection and is also often associated with some mutations in the ‘a’ determinant such as G130N, F134L, D144A, D144G, G145A, G145K, and G145R or failed hepatitis B immune globulin (HBIG) prophylaxis. It has also been reported that the naturally occurring mutation sM133T can create a novel N-linked glycosylation site in the viral envelope proteins (Ito et al., 2010). The findings of the present study indicate the need for a large scale study that also includes HBsAg-negative patients in order to investigate occult HBV infections in the CAR population, as has been suggested previously (Komas et al., 2013).

In addition to the three IEMs identified, several surface protein mutations were identified (sP56Q, sT57I, sN59S, sP62L, sI110L, sS140T, sE164G, sS207N, and sL216*), which have also been reported in previous studies (Mello et al., 2011; Lin et al., 2013; Munshi et al., 2017; Qin and Liao, 2018). The most frequently occurring surface protein mutation was sN59S, harbored by 46/51 isolates. This was previously reported to affect T helper/cytotoxic T lymphocyte (TH/CTL) inter-epitopes (Lin et al., 2013; Qin and Liao, 2018). Mutations sP56Q and sP62L, harbored by two HBeAg-negative patients, have previously been reported to be associated with an increased risk of hepatocellular carcinoma (HCC) (Lin et al., 2013; Munshi et al., 2017; Qin and Liao, 2018); the serological analysis identified 102/118 patients in this study to be HBeAg-negative. Indeed, the loss of HBeAg has been reported to be a marker associated with the end of active viral replication and improvement in HBV disease (Lazarevic, 2014). Currently, the increasing mutation in the HBV genome is making serologically negative HBeAg results uncertain in some African countries (Olinger et al., 2006; Belyhun et al., 2018; Mokaya et al., 2018). HBeAg, hepatitis B core antigen (HBcAg), and the core-related protein p22 are all proteins produced by the pre-core/core HBV gene and they share the same 149 amino acid sequence; therefore, quantification of the HBV core-related antigen (HBcrAg) has been shown to be more accurate (Hadziyannis and Laras, 2018) than HBeAg serology alone. Co-infection with HDV was found in 23 of the 118 patients, a prevalence similar to that reported previously in CAR (Komas et al., 2018).

### HBV polymerase mutants

This study identified several known polymerase mutations (rtL91I, rtM129L, rtW153R, rtS213T, rtV214A, rtN238T, rtN248H, and rtI269L), recorded in many previous studies (Locarnini, 2008; Abdelnabi et al., 2014; Gomes-Gouvêa et al., 2015; Zhang and Ding, 2015; Kim et al., 2017; Yamani et al., 2017; Choi et al., 2018; Shaha et al., 2018; Ababneh et al., 2019). The most frequently identified polymerase mutation was rtL91I, harbored by six isolates. The study did not detect any drug resistance mutations among the polymerase mutants recorded. Since the records of the HBsAg-positive patients at Institut Pasteur de Bangui indicated that they had not received any nucleoside/nucleotide analog (NA) treatment, the probability of detecting HBV drug resistance was expected to be very low: as discussed before in California, Brazil, and elsewhere, HBV drug resistance is very rare in treatment-naïve patients (Nguyen et al., 2009; Abdelnabi et al., 2014; Gomes-Gouvêa et al., 2015; Rugieri Pacheco et al., 2017).

It is important to note that there is currently no therapeutic support of chronically infected HBV patients in the CAR. Most of the patients used traditional medicine to overcome this issue. HBV patients received treatment in the case of HIV/HBV co-infection, and a very limited number of patients who had sufficient finance to support their treatment also received HBV treatment. This is one of the challenges for investigating HBV drug resistance properly, unless HIV/HBV co-infected patients are included. The inclusion of HIV/HBV co-infected patients is thus recommended for further investigations into HBV drug resistance in the CAR.

### HBV genotypes and serotypes

Although the present study identified two HBV genotypes (A and E), HBV genotype E was the most identified genotype and this remains the dominant strain circulating in the CAR. This is in agreement with previous studies showing genotype E to be dominant in Western and Central Africa (Bekondi et al., 2007; Komas et al., 2013; Kramvis, 2014). In fact, HBV genotype E has low diversity and emerged more recently, within the last 200 years (Kramvis, 2014). The two subgenotype A1 isolates (Pol33 and Pol40) harbored 14 and 13 amino acid exchanges, respectively, in the polymerase gene, among which nine (rtN122H, rtN124H, rtM129L, rtS137T, rtW153R, rtV163I, rtS256C, rtT259S, and rtI269L) were not present in genotype E (of the 49 isolated genotype E, 22 did not have any amino acid exchanges in the polymerase gene), showing that genotype A circulating in CAR is likely to undergo mutations that will affect the diagnosis, as well as the prevention and control measures for HBV in the CAR population.

In addition to genotype classification, this study serotyped the isolated strains. Two serotypes were identified: ayw4 accounted for all genotype E and ayw1 accounted for all subgenotype A1. This result is not surprising, as the HBV serotype is strongly associated with the genotype (Kramvis et al., 2008). However, this information is still useful, since data on isolated HBV from CAR are limited.

### Conclusions

Genotype E, presenting less genetic variability, remains the dominant strain circulating in the CAR. This study identified potential IEMs and several polymerase mutants among the limited population of patients attending Institut Pasteur de Bangui, confirming the hypothesis of circulating HBV IEM strains in the CAR population. The results of this study raise awareness of the need for further studies to be conducted on a large scale, including patients under treatment, in order to better understand IEMs of HBV for improved disease prevention and control strategies in the CAR.
