# Supplementary material for: Detection of circulating hepatitis B virus immune escape and polymerase mutants among HBV-positive patients attending Institut Pasteur de Bangui, Central African Republic
Source: Int J Infect Dis. 2019 Nov 1;90:138–44. doi: 10.1016/j.ijid.2019.10.039 (PMC6912157; doi:10.1016/j.ijid.2019.10.039)
Supplement: Supplementary file 1 [file IJID-2019-J-IJID-2019-10-039-s1.docx]

Supporting material: Mutations identified in surface antigen and polymerase/reverse transcriptase of the 51 isolated HBV

| **Sample ID** | **Genotype** | **Surface antigen mutation** | **Polymerase/RT mutations** |
| --- | --- | --- | --- |
| **Pol 1** | E | T63I |  |
| **Pol 7** | E | S204R, Y206F | S117P, S213T, L220I, M267L |
| **Pol 8** | E | N59S, E164G | L91I |
| **Pol 11** | E | N59S |  |
| **Pol 16** | E | N59S |  |
| **Pol 17** | E | N59S |  |
| **Pol 18** | E | N59S |  |
| **Pol 28** | E | N59S | L115V |
| **Pol 31** | E | N59S | L115V |
| **Pol 32** | E | N59S |  |
| **Pol 33** | A(A1) | Y100C, I110L, K122R, F134I, V190A, Y206H, S207N, L216* | R110G, N118T, N122H, N124H, M129L, S137T, V142D, W153R, V163I, V214A, N238T, S256C, T259S, I269L |
| **Pol 34** | E | N59S | L91I, S135A, K270N |
| **Pol 38** | E | N59S |  |
| **Pol 39** | E | N59S, M133T, P214L, C221Y, Y225* | H234N |
| **Pol 40** | A(A1) | K122R, S207N | N122H, N123D, N124H, M129L, S137T, W153R, V163I, S246T, N248H, I253K, S256C, T259S, I269L |
| **Pol 42** | E | N59S, N207T, I208T, V224A | S109P, I122L, Q215H |
| **Pol 44** | E | N59S |  |
| **Pol 45** | E | N59S, A128V | Q125K, P130S, N248H |
| **Pol 47** | E | N59S, R79H, F83S | L91I, N118D, N238H, M267L |
| **Pol 48** | E | N59S | R110G, S223A, N238H, M267L |
| **Pol 50** | E | N59S | L91I |
| **Pol 57** | E | N59S, L109P |  |
| **Pol 58** | E | S55F, N59S, I68T, L127I, P203R | L91I, S135H, S223A, N238H, M267L |
| **Pol 59** | E | N59S, G130S | L115V, R138K, N139H, E263D |
| **Pol 60** | E | N59S, W74L, L209S |  |
| **Pol 62** | E | N59S | S223A |
| **Pol 64** | E | N59S, V177A | S223A, M267L |
| **Pol 65** | E | N59S, V177A | S223A, M267L |
| **Pol 68** | E | N59S |  |
| **Pol 76** | E | N59S | S223A |
| **Pol 78** | E | N59S | S223A |
| **Pol 80** | E | N59S | S223A, M267L |
| **Pol 87** | E | N59S |  |
| **Pol 88** | E | P56Q, T57I, N59S, P62L, T189I, L209W, I213T, P217L, V224A |  |
| **Pol 91** | E | N59S, P211H | S117P, N139D |
| **Pol 93** | E | N59S |  |
| **Pol 94** | E |  | N248H, R266K |
| **Pol 100** | E | N59S | S223A, M267L |
| **Pol 101** | E | N59S |  |
| **Pol 102** | E | N59S |  |
| **Pol 103** | E | N59S | S223A |
| **Pol 105** | E | N59S | S223A, M267L |
| **Pol 106** | E | N59S |  |
| **Pol 107** | E | N59S | S223A |
| **Pol 108** | E | N59S, P203R |  |
| **Pol 110** | E | N59S, S140T | F148Y, R266T |
| **Pol 111** | E | N59S |  |
| **Pol 112** | E | N59S |  |
| **Pol 115** | E | N59S | L91I |
| **Pol 117** | E | N59S, Q101H, A184V |  |
| **Pol 118** | E | N59S, P203R |  |

*Sample ID is the particular identity assigned during the research to substitute the patient’s data. Each row gives respectively the information about the genotype, surface antigen mutations and polymerase mutations.*
